# Artificial selection increased body weight but induced increase of runs of homozygosity in Hanwoo cattle

**DOI:** 10.1371/journal.pone.0193701

**Published:** 2018-03-21

**Authors:** Kwondo Kim, Jaehoon Jung, Kelsey Caetano-Anollés, Samsun Sung, DongAhn Yoo, Bong-Hwan Choi, Hyung-Chul Kim, Jin-Young Jeong, Yong-Min Cho, Eung-Woo Park, Tae-Jeong Choi, Byoungho Park, Dajeong Lim, Heebal Kim

**Affiliations:** 1 Interdisciplinary Program in Bioinformatics, Seoul National University, Kwan-ak Gu, Seoul, Republic of Korea; 2 C&K genomics, C-1008, H businesspark, 26, Beobwon-ro 9-gil, Songpa-gu, Seoul, Republic of Korea; 3 Department of Agricultural Biotechnology, Seoul National University, Kwan-ak Gu, Seoul, Republic of Korea; 4 Department of Agricultural Biotechnology, Animal Biotechnology Major, and Research Institute for Agriculture and Life Sciences, Seoul National University, Seoul, Korea; 5 National Institute of Animal Science, Rural Development Administration, Wanju, Republic of Korea; Wageningen UR Livestock Research, NETHERLANDS

## Abstract

Artificial selection has been demonstrated to have a rapid and significant effect on the phenotype and genome of an organism. However, most previous studies on artificial selection have focused solely on genomic sequences modified by artificial selection or genomic sequences associated with a specific trait. In this study, we generated whole genome sequencing data of 126 cattle under artificial selection, and 24,973,862 single nucleotide variants to investigate the relationship among artificial selection, genomic sequences and trait. Using runs of homozygosity detected by the variants, we showed increase of inbreeding for decades, and at the same time demonstrated a little influence of recent inbreeding on body weight. Also, we could identify ~0.2 Mb runs of homozygosity segment which may be created by recent artificial selection. This approach may aid in development of genetic markers directly influenced by artificial selection, and provide insight into the process of artificial selection.

## Introduction

Artificial selection creates genetic signatures on a genome as well as alteration of phenotypes [1–3]. These genetic signatures refer to any types of sequence alteration which can be generated from selection process. For instance, extended linkage disequilibrium (LD), and reduced nucleotide diversity are examples of the genetic signatures [4, 5]. In the artificial selection process, one of the factors that give rise to such genetic signatures is inbreeding, which is the production of offspring from mating or breeding of individuals that are genetically close [6]. Breeders allow only certain individuals with desirable characteristics to reproduce over the course of decades resulting in intensive inbreeding [7].

Runs of homozygosity (ROH) refers to the contiguous homozygous segment in the genome of an individual [8]. During inbreeding, identical-by-descent tracts could be formed in the genome of an individual, which refers to ‘autozygous segment’. The homozygosity of the tract refers to ‘autozygosity’. Although ROH segment could include randomly created segments, when the length is long enough it has been commonly regarded as autozygous segment. One of the most important features of autozygous segments is that their length shortens over generations due to meiotic recombination [9]. For this reason, by controlling the length threshold, it is possible to obtain the autozygous segment which was created during the period we are interested in. This was recently demonstrated using simulated data [10]. Several studies have analyzed ROHs including the autozygous segments, to detect recent artificial selection signatures [11, 12]. The general process of artificial selection is accompanied by inbreeding and the degree of inbreeding increases as artificial selection progresses [13]. Therefore, it was also possible to investigate the change of autozygosity level as signatures of recent artificial selection by using genotype data which was accumulated for decades [13]. In addition to detecting the signature of artificial selection, ROH have been used as genetic markers for complex traits as well as Mendelian traits. For instance, ROH was suggested as the risk factor for schizophrenia in the human population [14] and used to detect inbreeding depression for two reproductive traits in the pig population [15].

Hanwoo cattle originated from natural crossbreeding between taurine and zebu cattle in Korea [16]. In earlier times of its 5000 years history, Hawoo was used as draft animal and was not artificially selected [17]. In the early 1900's, Hanwoo was selected by appearance traits including body type and hair color to determine the breed, following the guideline established by the government agency [18]. Since the establishment of selection purpose for beef cattle in 1963 by Livestock Industry Act (LIA), Artificial selection of Hanwoo commenced along with the development of AI [18]. In 1974, performance tests was developed to select cattle with superior characteristics as beef cattle [18]. In 1980s, progeny test, which is a method that tests cattle by examining the economic traits observed from their offspring, was introduced by Hanwoo Improvement Center (HIC) [18]. Since the introduction of the two tests, Hanwoo breeding program has expanded and bulls with the proven quality could be produced. In the current performance test, young bulls from 6 to 12 month of age are selected through genetic evaluations for yearling weight (YW) and marbling score (MS) [18]. Semen collected from the selected young bulls is then inseminated in cows whose lineage is known [18]. Progenies of the cows are weighed at 6, 12, 18 and 24 months of age, and harvested at 24 months of age to investigate carcass traits including carcass weight (CW), eye muscle area (EMA), back fat thickness (BF) and MS. Selection of proven bulls is then conducted, based on a selection index which is measured by using weighted breeding values for BF, MS and EMA [17]. Although the current breeding program of Hanwoo cattle has been established recently, their economic traits has improved overall. Especially, YW remarkably increased from 315.54 kg in 1998 to 355.06 kg in 2011, resulting in about 40 kg of improvement over 13 years [18].

For decades, Hanwoo breeding program has selected a subset of Hanwoo population, and the selected bulls have been used as breeding population through AI. Therefore, it is likely that there has been increase of inbreeding, considering similar cases of cattle population [19, 20]. In case of Hanwoo population, there was a report that the averaged inbreeding coefficients increased by 0.3% from 1997 to 2007, when a total of 1,123,162 cattle was investigated [21]. While the inbreeding is inevitable during artificial selection or breeding, it has also deleterious effect on reproductive or production traits of individual [22] due to increased homozygosity at loci carrying rare recessive deleterious alleles or exhibiting overdominance [23, 24]. In Hawoo, there was a report that inbreeding affected body weights at birth or at weaning in a negative way [25]. However, the degree of inbreeding has been known to be lower than other commercial cattle breeds so far [26]. For this reason, in Hanwoo population, it is important to control genetic diversity of the population to manage deleterious effect of inbreeding resulted from intensive artificial selection.

In this study, we aimed to investigate the changes of inbreeding in Hanwoo population during the decades of selection program, and identify genomic regions related to inbreeding induced by artificial selection and a trait (body weight). Especially, Hanwoo population used in this study has been intensively selected for decades to improve their economical traits including the body weight of a calf. Therefore, identifying the genomic regions affecting body weight during breeding program is important. In order to accomplish this, whole genome sequencing data of 126 cattle which were selected by a breeding program during ~20 years were generated. After defining autozygous segments on the whole genome data, we demonstrated the change of autozygous level in the population. Also, two statistical analyses was performed between three elements (artificial selection, autozygosity level, and body weight) using two regression models. Using the combined results of the two regression analysis, we attempted to demonstrate the relationship between these three elements.

## Materials and methods

### Ethics statement

No ethics statement was required for the collection of DNA samples. DNA was extracted either from artificial insemination bull semen straws or from blood samples obtained from the Hanwoo Improvement Center of the National Agricultural Cooperative Federation (HICNACF) with the permission of the owners. The protocol was approved by the Committee on the Ethics of Animal Experiments of the National Institute of Animal Science (Permit Number: NIAS2015-774).

### Sample information and whole genome sequencing

Blood samples for whole genome sequencing were obtained from 136 Korean beef cattle, (Hanwoo), 126 of which were bulls that were selected from HICNACF. The breeding program consisted of two steps; Performance and Progeny test of candidate bulls. In the first step, 66 of 900 bulls were selected by their weighted breeding values of economic traits, weight at 12 months, and marbling score. In the second step, 4,500 cows were inseminated with the semen from the selected bulls and 30 bulls were selected based on selection index which used weighted breeding values for BF, MS, and EMA of 800 male calves [18, 27, 28]. Our 126 bulls have been selected through this breeding program from 1998 to 2015. The unselected cattle (n = 10) reared in Hanwoo Research Institute in National Institute of Animal Science had never been involved and selected in Hanwoo breeding program.

Using DNA from the blood samples, we produced indexed shotgun paired-end (PE) libraries with approximately 500bp inserts using TruSeq Nano DNA Library Prep Kit (Illumina, USA) following standard Illumina sample-preparation protocol. Briefly, 200 ng of gDNA was fragmented by Covaris M220 (Woburn, MA, USA) resulting in a median fragment size of ~500 bp followed by end repair, A-tailing, and indexed adapter ligation (~125bp adapter). For the next step, the gel-based size selection was done in the range of 550 to 650 bp for the adapter ligated DNA and PCR amplification was performed for 8 cycles in the case of library. The size-selected libraries were analyzed by an Agilent 2100 Bioanalyzer (Agilent Technologies) to determine the size distribution and to check for adapter contamination. The resulting libraries were sequenced in Illumina HiSeq 2500 (2x125bp paired-end sequences) and NextSeq500 (2x150bp paired-end sequences) sequencer.

### Sequence read mapping and variant calling

A quality control for per-base quality of reads and removal of potential adaptor sequences was performed using fastQC [29] and Trimmomatic [30] software, respectively. Then, high quality sequence reads were mapped to the *Bos taurus* reference genome (UMD 3.1.78) using Bowtie2 [31] with default settings. A series of downstream processes were performed to improve the quality of called variants as well as sequence alignment: Picard tools (http://picard.sourceforge.net) was used to sort reads, remove potential PCR duplicates, and ensure the mate pair information of paired-end reads. SAMtools [32] was used to create index files for the reference and bam files. Genome Analysis Toolkit (GATK) [33] was used to conduct local realignment of sequence reads to correct misalignments aroused by small insertion and deletion. Also, base quality scores were recalibrated to obtain more accurate quality scores and correct the variation in quality with machine cycle and sequence context. Lastly, for variant calling and filtering step, “UnifiedGenotyper” and “SelectVariants” arguments implemented in GATK were used. High quality variants were retrieved by employing following criteria: The variants with 1) a Phred-scaled quality score < 30, 2) read depth < 5, 3) MQ0 (total count across all samples of mapping quality zero reads) > 4; or a 4) Phred-scaled P-value using Fisher’s exact test > 200 were filtered out to reduce false positive calls.

### Detection of ROH and autozygous segments

Using the information of genotypes provided by variant calls, ROH for each sample was identified using vcftools [34, 35]. As all of the genotypes were obtained from bulls, the homozygosity segments within only autosomal regions were considered. To ensure that the detected ROHs had not been generated by random events but by recent inbreeding, the proportion of ROHs in the population was investigated according to change of ROH length threshold. From this, the threshold of ROH length was set to 500kb, and ROHs shorter than 500kb were filtered out. We regarded the remaining ROHs as autozygous segments for the following downstream analysis (S1 Dataset). The proportion of these autozygous segments in UMD 3.1 reference genome (F_roh_) was calculated and comparison was made between groups by Wilcoxon’s rank sum test.

### Detection of inbreeding and selection signatures

Inbreeding coefficients of each individuals based on SNP (F_snp_) were estimated as follows. First, whole SNP data set was pruned using the option—indep-pairwise implemented in plink 1.9 [36] with three parameters (window size = 50, step size = 5, and r2 = 0.5). Then, individual F_snp_ was estimated from the data set using the option–het. LD between pairs of markers were assessed using plink 1.9 [36]. The r^2^ value was calculated between all pairs of SNPs within 20 kb (—r^2^ and—ld-window-kb parameters). Moving averages of the pairwise r^2^ were then carried out with 5-kb steps.

To detect recent selection signatures, we used SHAPEIT v2 [37] to infer the haplotype phase and impute missing alleles for the SNP data set generated after filtering out SNPs based on genotype missing rate > 0.05, and minor allele frequency < 0.01. Then, integrated haplotype score (iHS) was calculated with the Selscan [38] using the default settings except—maf 0.01 option. The raw iHS was standardized by ‘norm’ module implemented in Selscan with 100 frequency bins.

### Statistical analysis to identify candidate genomic region

To identify genomic regions associated with inbreeding induced by artificial selection, traits or both, an association test with regression analysis was performed. The unit for the association test was determined to be 10Mb bins, and Bos taurus autosome (BTA) were divided into 269 bins of 10Mb. After filtering out the bins where all samples had an equal ROH length, 264 bins were finally used for statistical analysis.

In the first step of regression analysis, an association test between artificial selection and ROH was performed for each bin (Analysis 1). The progress of artificial selection was represented by ‘KPN (Korean Proven Bull) number’. KPN number is a registration number given to a bull finally selected through the breeding program. The breeding program has been performed approximately once a year. Accordingly, KPN numbers are highly correlated to the birth years of the selected bulls (Spearman’s correlation test, ρ = 0.9992, p-value < 2.2e-16). The data including birth year records were not available for some of the samples and the exact time of birth was not necessary for the measure of relative progress of artificial selection. Thus, KPN number which is available in all samples was used instead of birth year. Consequently, we performed statistical analysis between ROH status and KPN number with the logistic regression model [13]. In the model, indicator of the ROH status (0: no ROH, 1: at least one ROH) and KPN number were considered as response and explanatory variables, respectively.

In the second analysis, the mixed effect model was used to analyze an association between ROH length and body weight for each bin (Analysis 2). In the model, ROH length and facility where the sample was raised from, were considered as fixed effects. To adjust background genetic effect on the model, genomic relationship matrix (GRM) generated by GCTA tool [39] was considered as random effect. Statistical test was performed using asreml and wald function implemented in ASReml-R package [40], where Body weight at 12 month was used as response variable. In these two analyses, we determined significance of the each bin with p-value < 0.01.

### Validation of ROH segments using additional dataset

To confirm the existence of ROH segments in candidate regions, we additionally generated whole genome sequencing data of 77 Hanwoo cattle whose KPN numbers range from 634 to 1017, by using the same sequencing procedure. The ROH segments of the additional data were detected by the identical process which was used for the original dataset.

## Results

### The increase of genome-wide autozygosities in 126 cattle under selection

As the length of ROH which is autozygous, decreases over generations, it is necessary to set a suitable threshold of ROH length in order to investigate the effects of recent inbreeding on a genome. Although previous studies on ROH of cattle population suggested several criteria for defining autozygous segments [11, 13], these were not applicable in our case due to the characteristics of Hanwoo population as well as differences in the data generation platform.

Instead, we first investigated the average count or length of ROH with controlling the threshold of ROH length in our data (S1 Fig). Average count and length of ROH at a 500kb threshold were ~46.13 and ~33Mb, respectively. Although it is difficult to directly compare the detected ROHs to a previous study [16], these count and length of ROHs were relatively higher. This can be partially explained by the difference of method used to detect ROH, which include smoothing of homozygosity. In addition, at a 500kb threshold, 125 of 126 cattle had at least one ROH above the threshold, and the number dropped drastically around 500kb. Considering these results, ROH length of at least 500kb was chosen for the downstream analysis; the ROH frequency in our population was large enough (~99%) to cover almost all individuals, and, at the same time, the length was maximum to enable statistical analysis using ROHs.

**Fig 1:**
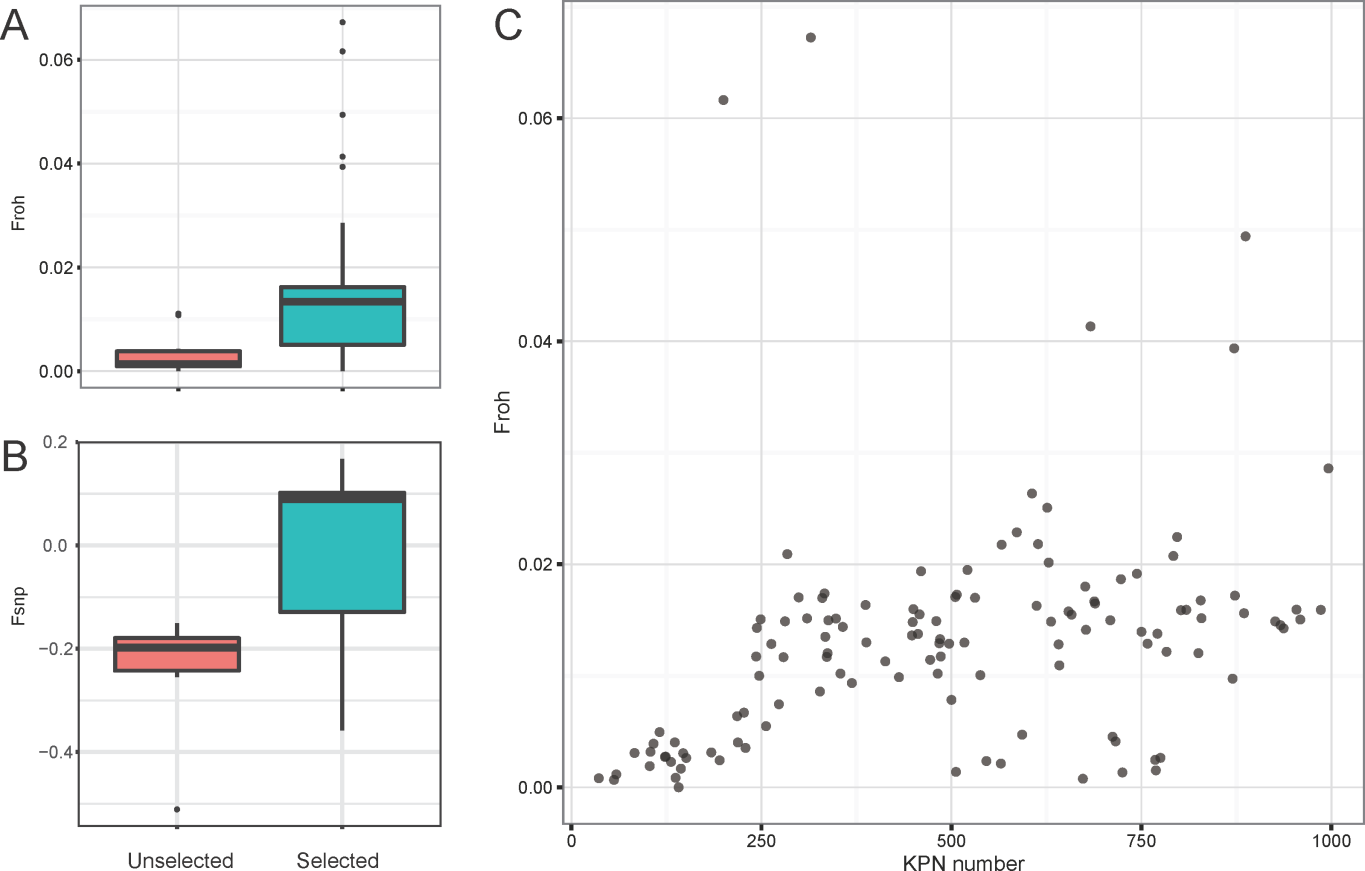
Individual genome-wide autozygosities (F_roh_). Comparison of (A) F_roh_ and (B) F_snp_ between selected (n = 126) and unselected (n = 10) cattle populations. Selected and unselected cattle populations were significantly different in both F_roh_ and F_snp_ (Wilcoxon rank sum test, p-value = 9.704e-05 and 2.979e-04, respectively). (C) Change of F_roh_ during the past ~20 years. KPN number was used instead of cattle birth year.

Although ROHs longer than 500kb occurred at least once in almost all individuals in our population, this alone does not suggest that 500kb ROHs were generated by recent inbreeding. Thus, it was necessary to show the difference between selected population that has gone through the inbreeding induced by artificial selection, and unselected population when the threshold was applied. For this, we used the genome data of unselected individuals which were processed in the same way as those of 126 selected individuals, and calculated individual genome-wide autozygosities (F_roh_) using ROHs following previously published protocol [8]. F_roh_ values of the unselected group were compared to those of 126 selected individuals. As expected, selected populations generally had a higher inbreeding coefficient than unselected (Fig 1A, Wilcoxon rank sum test, p-value = 9.704e-05). Unselected population had an average of 0.0034 F_roh_; on the other hand, average F_roh_ of the selected population was 0.0131. This difference was also observed when we generated the data by random sampling thousand times from 126 selected individuals to reduce bias due to sample size (S2 Fig). F_snp_ values between unselected and selected population also showed similar pattern (Fig 1B). From this result, we inferred that recent inbreeding had an influence on genome and F_roh_, and found that a threshold of 500kb could be sufficient to represent the recent inbreeding which our samples had undergone.

In the same context of comparison between selected and unselected cattle, the effect of breeding program over years was also investigated in selected cattle. During ~ 20 years, F_roh_ steadily increased with several outliers (Fig 1C). Additionally, the F_roh_ of cattle born earlier were close to zero, whereas most of cattle born later had F_roh_ close to 0.02. This demonstrates throughout ~20 years long breeding program, there was a ~2% increase of ROH at a genome-level. This gradual increase of inbreeding level has also been observed in a study using pedigree-based method [41].

### Genomic regions with increase of autozygosity during artificial selection

When genome-wide distribution of ROHs was investigated, there was variation of ROH length as well as change of ROH length among genomic regions (S3 Fig); changes of ROH length were calculated by subtracting the mean length of one population from the other divided by KPN number. Mean length of ROHs ranged from 0.0Mb to 0.44Mb, and the change of ROH mean length ranged from -0.31Mb to 0.52Mb. This genome-wide heterogeneity of ROH allowed genome-wide mapping analysis, which need the prerequisite that ROHs are not evenly distributed on a genome. In addition to this, most of bins gained ROHs rather than losing them (S3 Fig), which is consistent with the change of individual autozygosity according to KPN number (Fig 1C). Consequently, there were discrepancies in rates of inbreeding across a genome, however, the overall tendency was toward an increment of ROHs during artificial selection.

On the basis of ROH heterogeneity, we first tried to identify genomic regions whose ROH significantly increased or decreased throughout ~20 years of the breeding program. When ROHs within each bin were fitted by the model (Analysis 1), in 225 of total 264 bins, coefficients were positive (S4 Fig). This again demonstrated that in most of regions in genome, ROH increased even though the rates of increase were heterogeneous among the regions. Similar tendency was observed in nominally significant bins (p-value < 0.05). For the result of statistical test, there were 8 bins (p-value < 0.01) which were statistically significant, with coefficients ranging from 0.00234 to 0.00542 (Table 1). Of 8 bins, only one bin overlapped with 16 ROHs in a previous study [16] using whole genome sequencing of Hanwoo bull. This ROH segment were specific to Hanwoo breed compared to Black Angus and Holstein. The ROH might have been created during recent Hanwoo breeding program where the population was not outbred with other breeds (S5 Fig).

**Table 1:** Candidate regions associated with years. Statistical test using Analysis 1 was performed. Only the regions with Pvalue less than 0.01 are shown.

BTA	Start	End	Coefficient	r^2^	Pvalue
1	130,000,001	140,000,000	0.00250	0.06192	0.00551
2	70,000,001	80,000,000	0.00235	0.05965	0.00209
6	80,000,001	90,000,000	0.00245	0.06217	0.00318
7	40,000,001	50,000,000	0.00294	0.07861	0.00436
9	1	10,000,000	0.00234	0.05587	0.00620
16	1	10,000,000	0.00542	0.18820	0.00092
18	30,000,001	40,000,000	0.00312	0.08871	0.00205
25	30,000,001	40,000,000	0.00327	0.08881	0.00674

**Fig 2:**
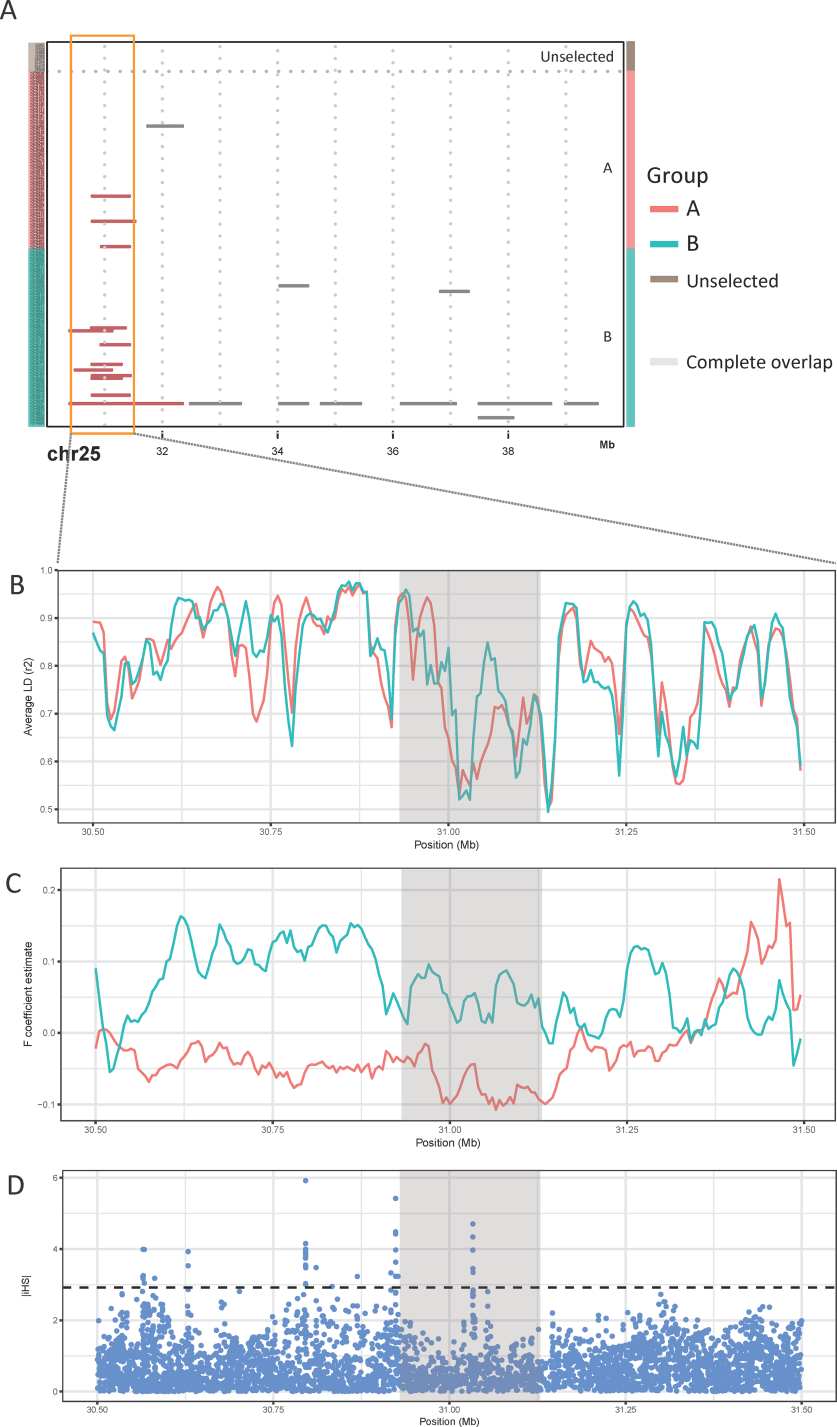
Signatures of inbreeding at the candidate region in BTA 25. (A) Distribution of ROH segments in the candidate region. “Complete overlap region” refers to the genomic regions that have the maximum number of samples which have at least one ROH segment. (B) Inbreeding signatures of candidate region are presented by Average LD and F coefficient. “Complete overlap region” are shaded in grey. Unselected individuals, Group A (Individuals with KPN≤486), and Group B (Individuals with KPN>486) are represented by dark brown, red and green color, respectively.

To gain deeper insight into the 8 regions identified above, we investigated the distribution of ROH segments (S6 Fig). We found that one of them (BTA 25, 30~40Mb) showed increasing pattern at a narrow region. 15 individuals contained at least one ROH segment in this region, and 12 segments among them shared ~0.2Mb region (BTA 25, 30,931,767~31,129,826) (Fig 2A). When we separated total population into two groups (Group A: KPN≤486, and Group B: KPN>486), Group B had more ROH segments than Group A, and Average LD and F coefficients of Group B was higher than Group A (Fig 2B). These measures of inbreeding suggest that in this particular region, there was an increase of inbreeding induced by artificial selection. Moreover, strong selection signature (|iHS| > 3.623, as highest 1% of all |iHS| values at genome-wide level) was detected in this region, especially in the overlapped ROH segment (Fig 2D). In a recent study, significant correlation between the EHH-based selection signature and actual trend in haplotype frequencies was demonstrated [42]. Similarly, we inferred that strong selection signature based on EHH could be the evidence of recent artificial selection contributing to change of ROH. The artificial selection causes sequence alteration, which in turn creates the inbreeding signature presented in this region. The existence of ROH segments in this region was also confirmed using additional dataset (S6 Fig).

### Lack of significant influence of recent inbreeding on body weight

**Fig 3:**
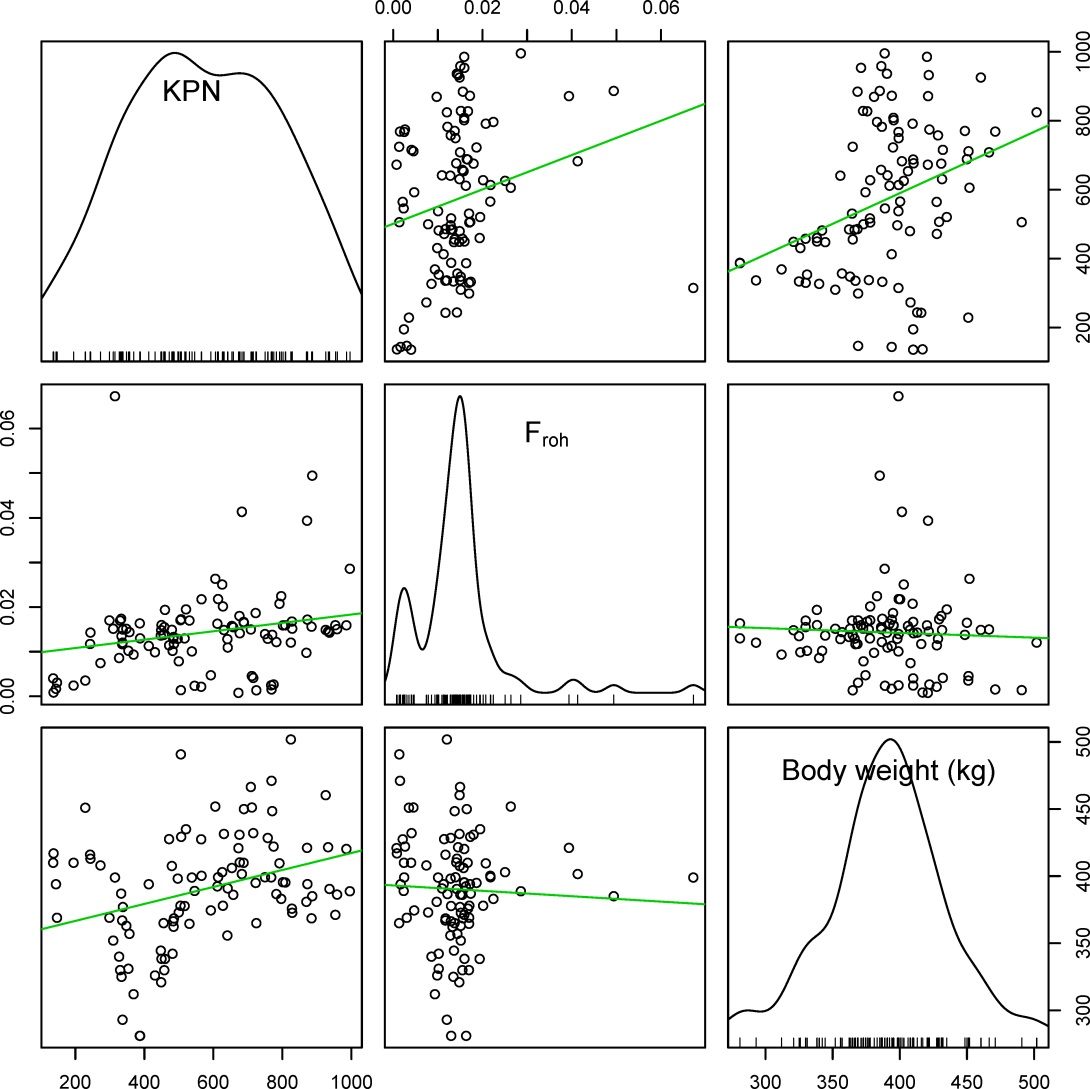
Scatterplots for KPN, F_roh_, Body weight. Correlation between each elements was tested by spearman’s method. KPN vs F_roh_: ρ = 0.46697, p-value = 5.203e-08; F_roh_ vs Body weight: ρ = -0.03930, p-value = 0.69900; KPN vs Body weight: ρ = 0.33921, p-value = 0.00059.

At a genome-wide level, there was no significant relationship between body weight and genome-wide F_roh_ (Fig 3, Spearman’s correlation test, ρ = -0.0393, p-value = 0.699). Instead, the body weight steadily increased during ~20 years, although F_roh_ steadily increased for the period (Fig 1C). Consequently, there was little influence of F_roh_ on body weight at an individual level. According to records for Hanwoo breeding program, there was indeed a gradual increase of weight for the decades [23] similar to our data (Fig 3). If we assume that F_roh_ represents the inbreeding level of an individual [8, 43], this suggests that although Hanwoo has experienced inbreeding pressure induced by artificial selection in ~20 years, the negative effect of inbreeding had less influence on an individual’s body weight.

When we focused on particular regions of 10Mb length, the regions with fitted models showing positive coefficient (Analysis 2) was almost equivalent to that of negative coefficient unlike the previous result (S4 Fig). However, the regions with negative coefficients are dominant if we look at the most nominally significant bins (p-value < 0.05). This discrepancy indicates that there are regions where ROHs have negative correlation with body weight. The 7 candidate regions associated with body weight are shown in Table 2 (p-value < 0.01). All of these regions were found to have a negative correlation between ROH length and body weight. Among these regions, 4 contain at least one body weight QTLs (body weight, or body weight gain) according to animal QTLdb [44]. Increase of 1 base pair of the ROHs in these regions corresponded to only 0.00002 body weight loss on average. Although ROHs located in these regions have negative correlation with body weight, it does not imply that the actual body weight of an individual will decrease due to these ROH. The actual trend of body weight of Hanwoo is towards an increase, and effect of the ROHs was one of many factors affecting body weight.

**Table 2:** Candidate regions associated with cattle body weight. Statistical test using Analysis 2 was performed. Only the regions with Pvalue less than 0.01 are shown.

BTA	Start	End	Coefficient	Pvalue	Body weight QTL
2	100,000,001	110,000,000	-0.00005	0.00076	0
3	90,000,001	100,000,000	-0.00002	0.00366	2
5	50,000,001	60,000,000	-0.00003	0.00037	1
7	70,000,001	80,000,000	-0.00002	0.00034	14
15	50,000,001	60,000,000	-0.00002	0.00451	0
16	40,000,001	50,000,000	-0.00001	0.00634	12
27	20,000,001	30,000,000	-0.00002	0.00441	0

### Signatures of selection detected by integrated haplotype score (iHS)

To detect loci under recent selection, we independently calculated iHS as a measure of selection. In each non-overlapping windows of 100kb, a proportion of SNPs with |iHS| > 2 was calculated (In this step, windows containing less than 10 SNPs were dropped). Then, we considered the windows with empirically highest 1% of the proportion (0.3623) to be candidates for containing selective sweep [45]. As a result, we could identify 250 windows with selective sweeps (S7 Fig). Of 8 regions in which ROH has increased significantly, 4 regions showed selective sweep signature, and 5 of 7 regions which were associated with body weight displayed selective sweep signature.

The candidate region in BTA 25 were not significantly affected by selective sweep. They showed relatively moderate signals (The proportion of SNPs with |iHS| > 2: 0.0432, and 0.0417 respectively). However, when we locally investigated the regions, we could find strong selection signals, especially in the overlapped ROH segment (Fig 2D).

## Discussion

In this study, we traced the change of inbreeding in cattle population under artificial selection, and observed increase of inbreeding for the decades of period. Also, we could suggest candidate regions which showed significant increase of inbreeding. However, we could not suggest strong evidences for the relation of the candidate regions produced by artificial selection to the increment of body weight. Indeed, there was no statistically significant regions shared by both of regression analyses (Analysis 1, and Analysis 2). Individual body weight has increased, even though the degree of inbreeding, which lowers fitness-related characters in many species [24] has also increased for decades of artificial selection, and the inbreeding of most candidate regions showed weak negative correlation with body weight. This might mean that the increment of inbreeding at genome-wide level or specific region did not have large impact on body weight.

Hanwoo breed has a short history of artificial selection compared to well-known commercial breeds such as Angus and Holstein [17, 18]. There has also been several evidences that the Hanwoo population has a lower degree of inbreeding than other commercial breeds [46, 47]. Therefore, it might be possible that the negative effect of inbreeding is not as high as the positive effect of the breeding program to cause deleterious effects on body weight in the Hanwoo population. Moreover, for weight/growth traits including body weight, it has been shown that there is less influence of inbreeding than other traits related to reproduction or production [48]. As a result, we inferred that either insufficient inbreeding load or characteristics of body weight trait could be the reason why increment of inbreeding did not have large effects on body weight.

In addition to investigation of inbreeding increment, we independently calculated iHS as a measure of recent selection. As a result, we could identify 250 windows with selective sweeps. However, the 250 windows could not include all of the previously identified regions with significant increase of autozygosity. Since inbreeding affects all loci equally and genetic drift changes frequency of loci randomly, inbreeding may not induce LD between neighboring loci, whereas selection will drive linked alleles to high frequency [49]. Selection signatures based on LD could not therefore capture all the actual change of ROH. Moreover, we investigated selection signatures for the entire population, which might result in more difference in the two analyses.

In previous studies, the patterns of ROH was investigated, in which ROHs of particular length in Hanwoo were compared to those in other commercial breeds [16, 50]. However, the purpose of this study was to demonstrate the change of ROH within particular population. The threshold for autozygous segments, therefore, needs to be set according to the characteristics of our population. Furthermore, the difference of marker density produced the difference of power to detect ROH that are IBD [51]. It is, therefore, expected that there are considerable differences in ROH detected from SNP chip data and sequencing data. Consequently, we set the threshold length of ROH adjusted to our data, unlike that of most previous studies, in which SNP chip data of commercial breeds were employed.

Here, we attempted to identify the direct connection between a genetic marker, breeding program and a trait. In past efforts to develop genetic markers in livestock, the main focus has been to find genetic markers significantly associated with economic traits. This approach could be promising in livestock industry if we have access to the tools that can directly manipulate genomic sequences with high resolution. However, there has been no such tool in livestock industry. Instead, artificial selection or breeding program have been widely used to indirectly modify genome sequences. Therefore, the chances of developing useful applications of the genetic markers are expected to be higher if breeding strategy as well as the trait are considered to identify those markers.

Artificial selection often leads to inbreeding [22], thus there have been many efforts to manage rates of inbreeding [52, 53]. The Hanwoo population used in this study has been known to have a relatively low inbreeding coefficient and larger effective population size than other commercial breeds such as Holstein [27, 54]. Howe**v**er, the degree of inbreeding in Hanwoo population rapidly increased in recent years, which has created an urgent need for the control of inbreeding rates [54]. Moreover, most Hanwoo calves are usually produced by artificial insemination which uses semen from a few selected bulls. For this reason, inbreeding of few bulls could affect the whole Hanwoo population. The approaches used in this study are advantageous for monitoring the change of inbreeding through breeding process along with inbreeding depression related to specific traits. This will be especially useful when investigating population of certain breed as Hanwoo.

## Conclusions

In this study, we generated whole genome sequencing data of 126 cattle under artificial selection, and observed increase of inbreeding during the decades of period. We showed that the increment of inbreeding through artificial selection did not have large impact on body weight for the decades, and identified a ~0.2Mb candidate ROH segment created by recent breeding program.

## Supporting information

S1 Dataset Detected entire ROHs longer than 500kb. (XLSX)

S1 Fig Mean count, length, and frequency of ROH in 126 cattle according to the change of ROH threshold. ROH threshold was controlled from 0 to 2000kb with 1000kb as a unit.
(TIFF)

S2 Fig Distribution of Mean F_roh_ and p-values of 1000 data sets generated by sampling 10 individuals from selected population iteratively. Fr_oh_ values for each data set were averaged, and p-values were calculated by Wilcoxon’s rank sum test between 10 selected individuals and unselected individuals (n = 10). Note that the vertical red line indicates Mean F_roh_ and p-value of unselected population, respectively.
(TIFF)

S3 Fig Genome-wide distribution of ROH in 126 cattle. (A) Distribution of ROH mean length in 10Mb bin. (B) Frequency of ROH longer than 500kb in 10Mb bin. (C) Change of ROH mean length when comparing ROH mean length of two groups (Group A: KPN≤486, and Group B: KPN>486).
(TIF)

S4 Fig Direction of regression coefficients in two association test (Analysis 1, and Analysis 2). (A) Bin counts according to their direction of regression coefficients in association test between artificial selection and ROH (Analysis 1). (B) Bin counts according to the direction of regression coefficients in association test between ROH and body weight (Analysis 2).
(TIF)

S5 Fig ROH length of each individuals in a bin (BTA2:70,000,001~80,000,000) overlapped with a previous study [16]. X axis indicates the individual ID sorted by their KPN number, and Y axis indicates ROH length in Mb.
(TIF)

S6 Fig Distribution of ROH segments in candidate regions with validation dataset. Y axis indicates the individual ID sorted by their KPN number with increasing order, and X axis indicates coordinates on UMD 3.1 reference genome. ROH segments in original dataset (n = 136), and in validation dataset (n = 77) are marked by grey and orange color, respectively. Note that the first 10 individuals is “unselected population” without KPN numbers.
(TIF)

S7 Fig Selective sweep regions identified by integrated haplotype score (iHS). The horizontal red line indicates top 1% proportion of SNPs with |iHS| > 2 in a 100kb window.
(TIFF)
