# Supplementary figures and images for: Artificial selection increased body weight but induced increase of runs of homozygosity in Hanwoo cattle
Source: PLoS One. 2018 Mar 21;13(3):e0193701. doi: 10.1371/journal.pone.0193701 (PMC5862439; doi:10.1371/journal.pone.0193701)

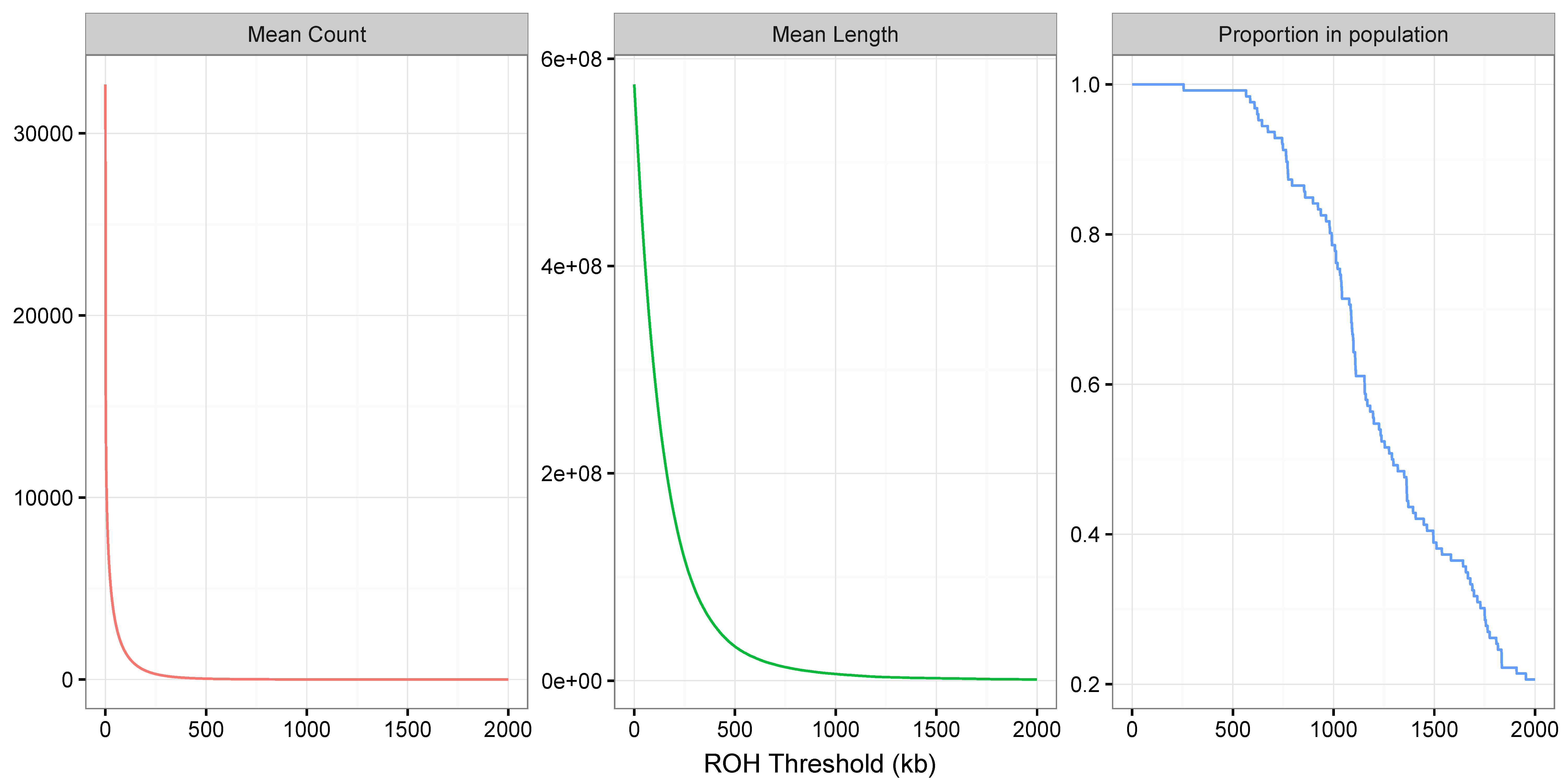

Supplement: S1 Fig — ROH threshold was controlled from 0 to 2000kb with 1000kb as a unit. (TIFF) [file pone.0193701.s002.tiff]

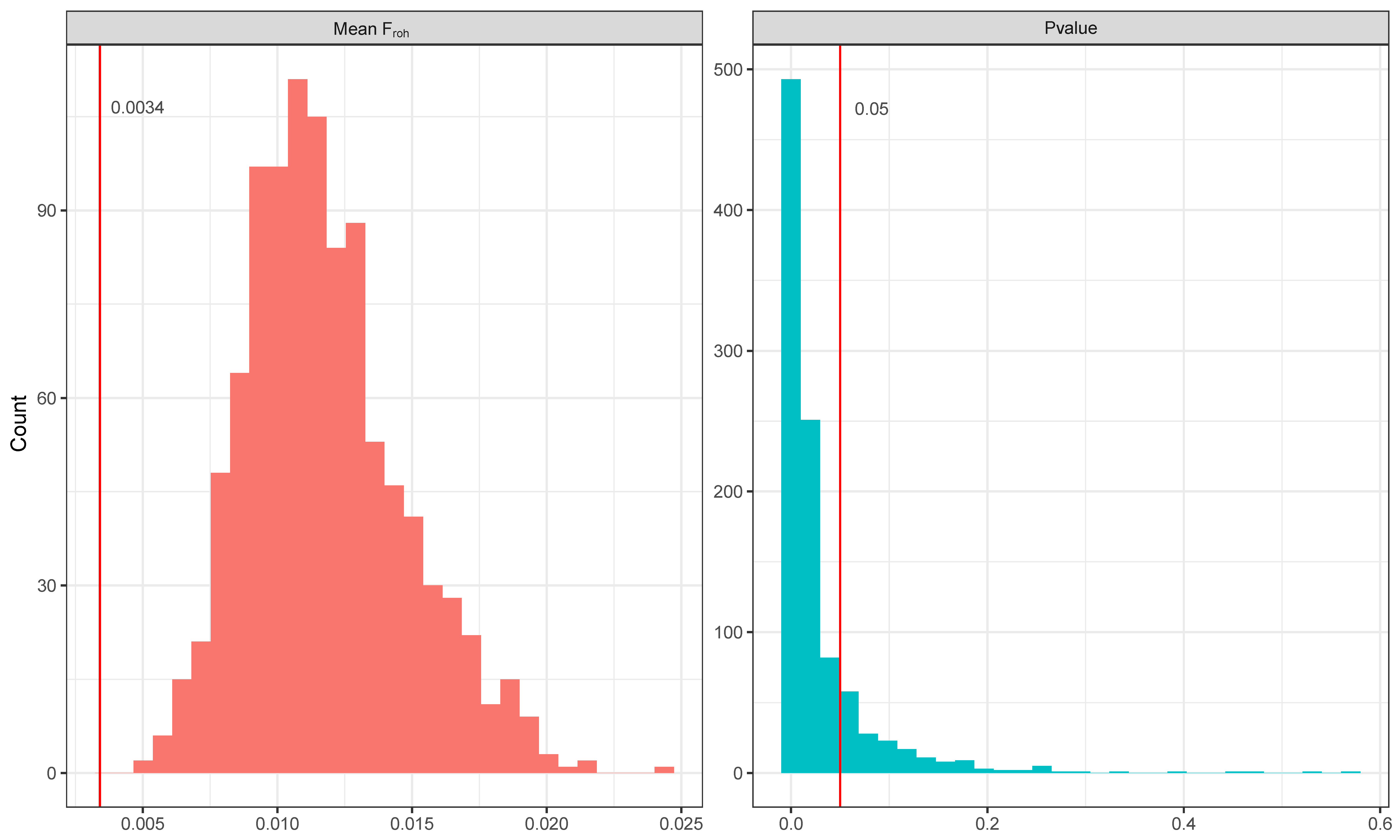

Supplement: S2 Fig — Froh values for each data set were averaged, and p-values were calculated by Wilcoxon’s rank sum test between 10 selected individuals and unselected individuals (n = 10). Note that the vertical red line indicates Mean Froh and p-value of unselected population, respectively. (TIFF) [file pone.0193701.s003.tiff]

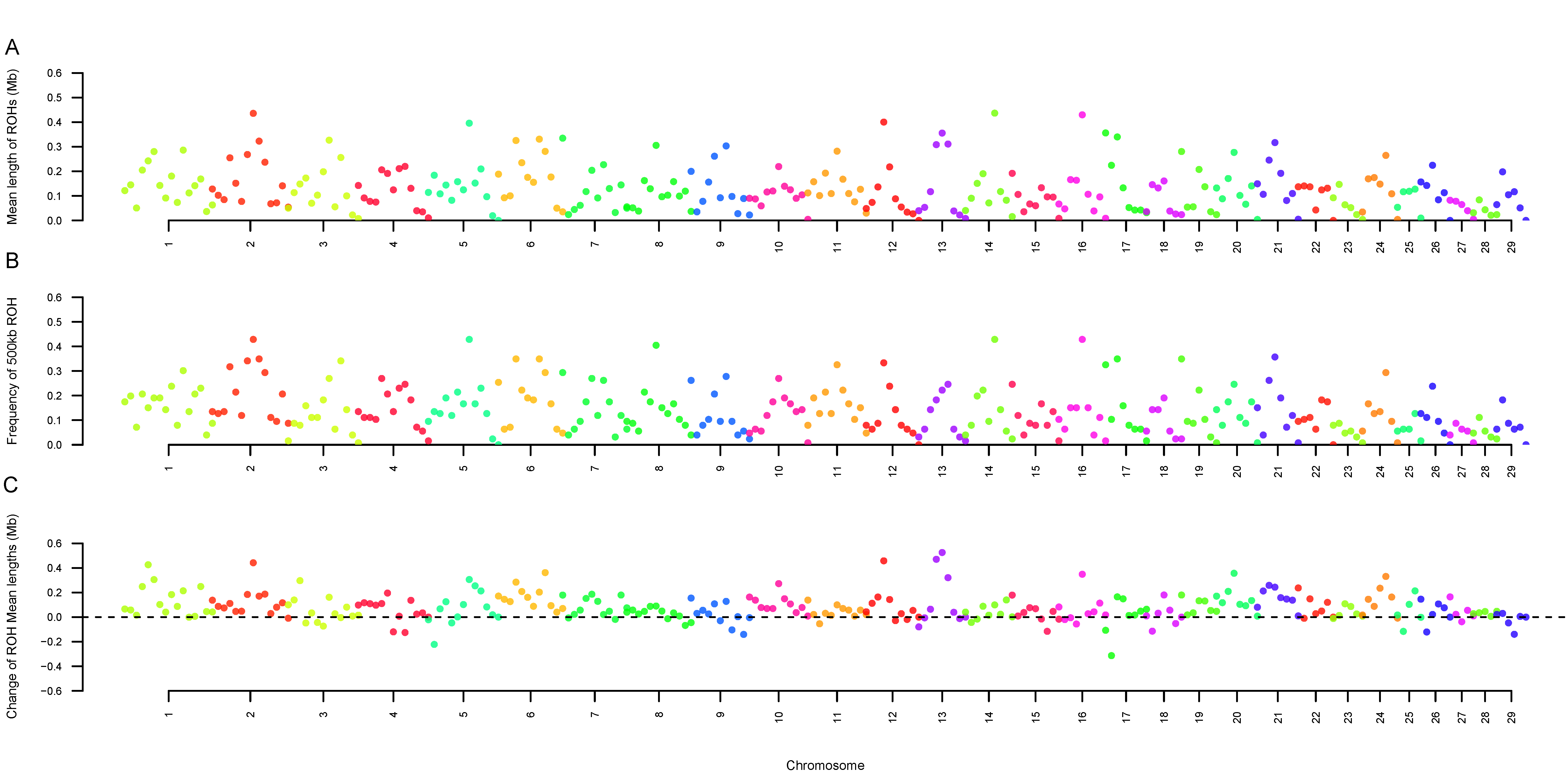

Supplement: S3 Fig — (A) Distribution of ROH mean length in 10Mb bin. (B) Frequency of ROH longer than 500kb in 10Mb bin. (C) Change of ROH mean length when comparing ROH mean length of two groups (Group A: KPN≤486, and Group B: KPN>486). (TIF) [file pone.0193701.s004.tif]

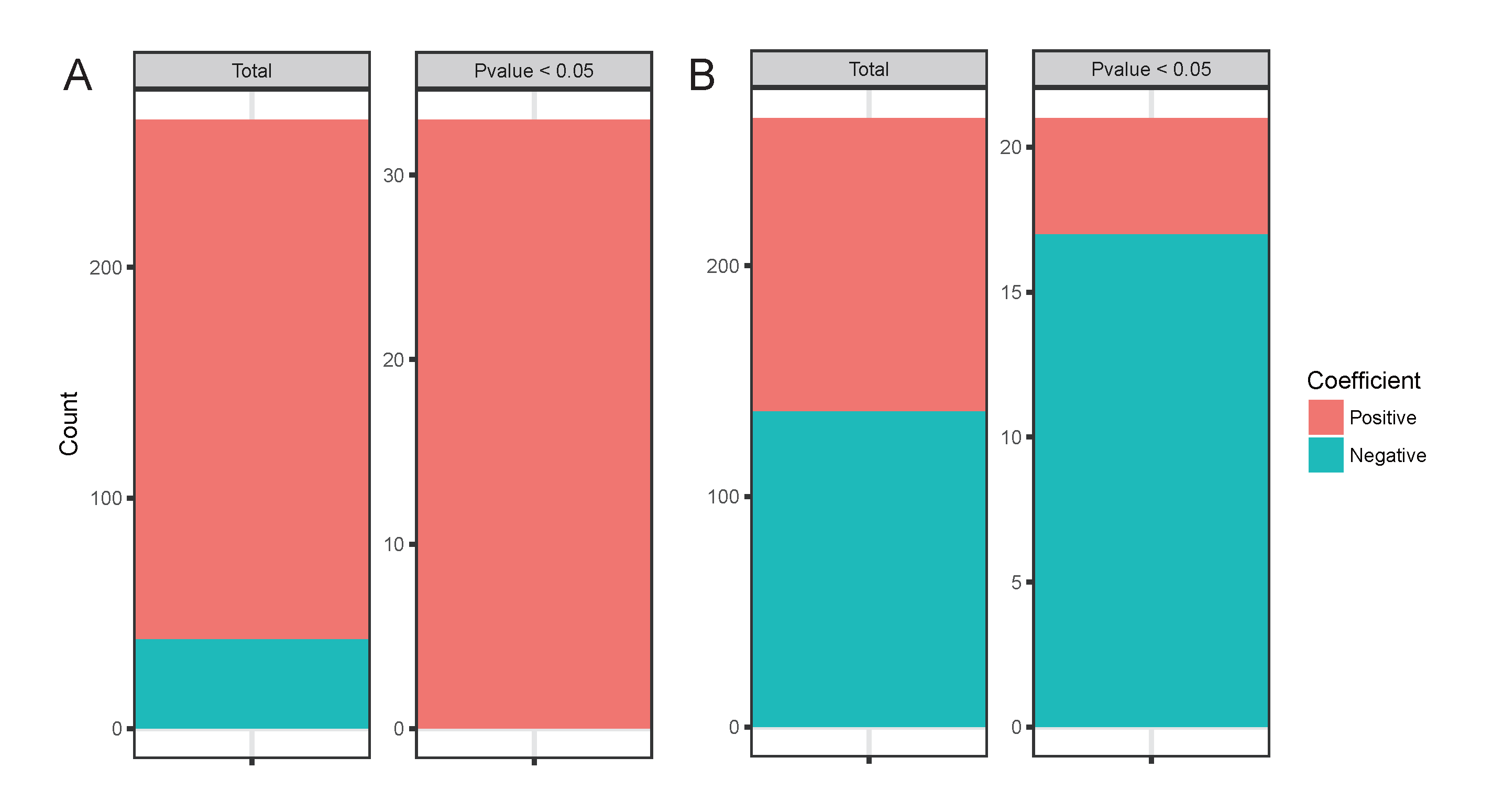

Supplement: S4 Fig — (A) Bin counts according to their direction of regression coefficients in association test between artificial selection and ROH (Analysis 1). (B) Bin counts according to the direction of regression coefficients in association test between ROH and body weight (Analysis 2). (TIF) [file pone.0193701.s005.tif]

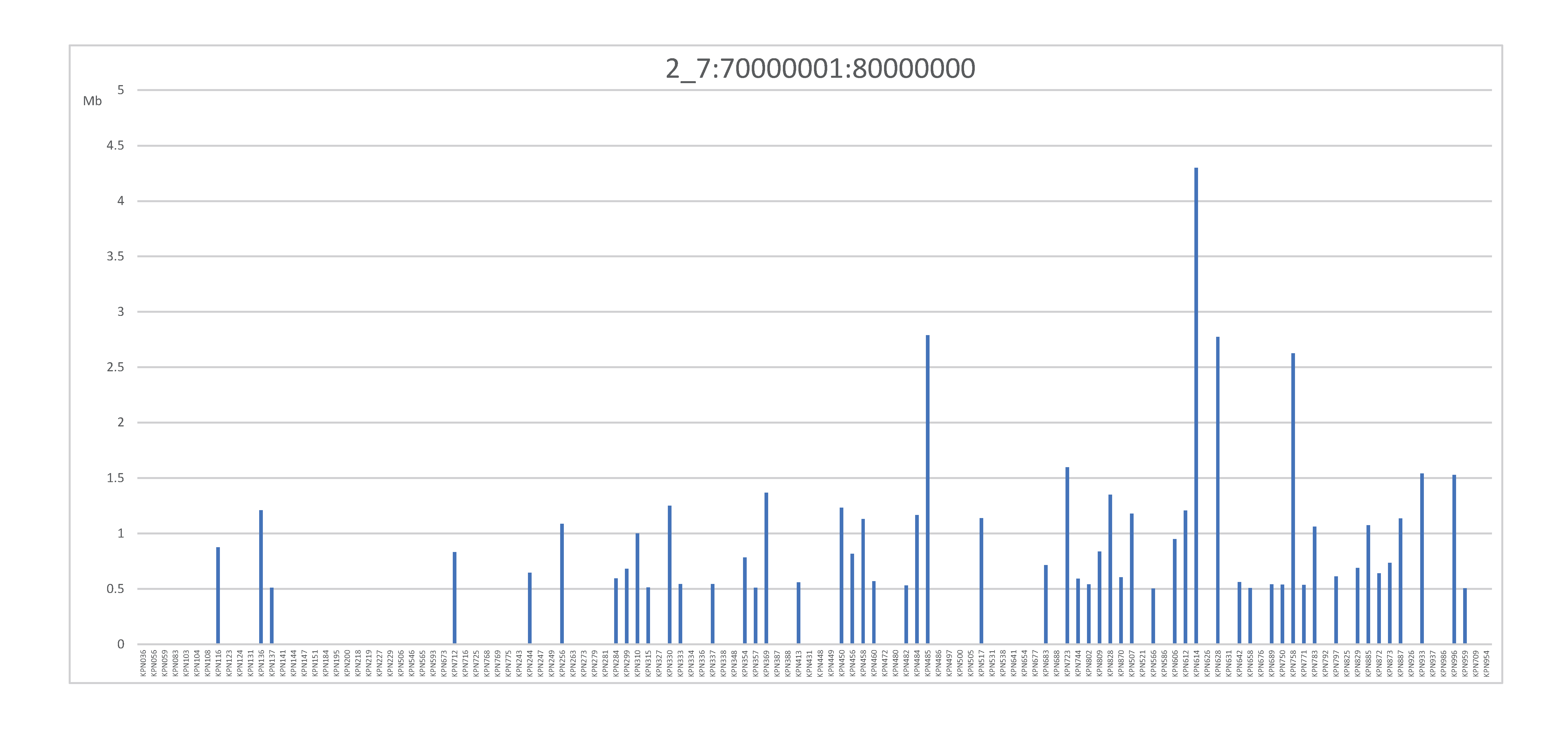

Supplement: S5 Fig — X axis indicates the individual ID sorted by their KPN number, and Y axis indicates ROH length in Mb. (TIF) [file pone.0193701.s006.tif]

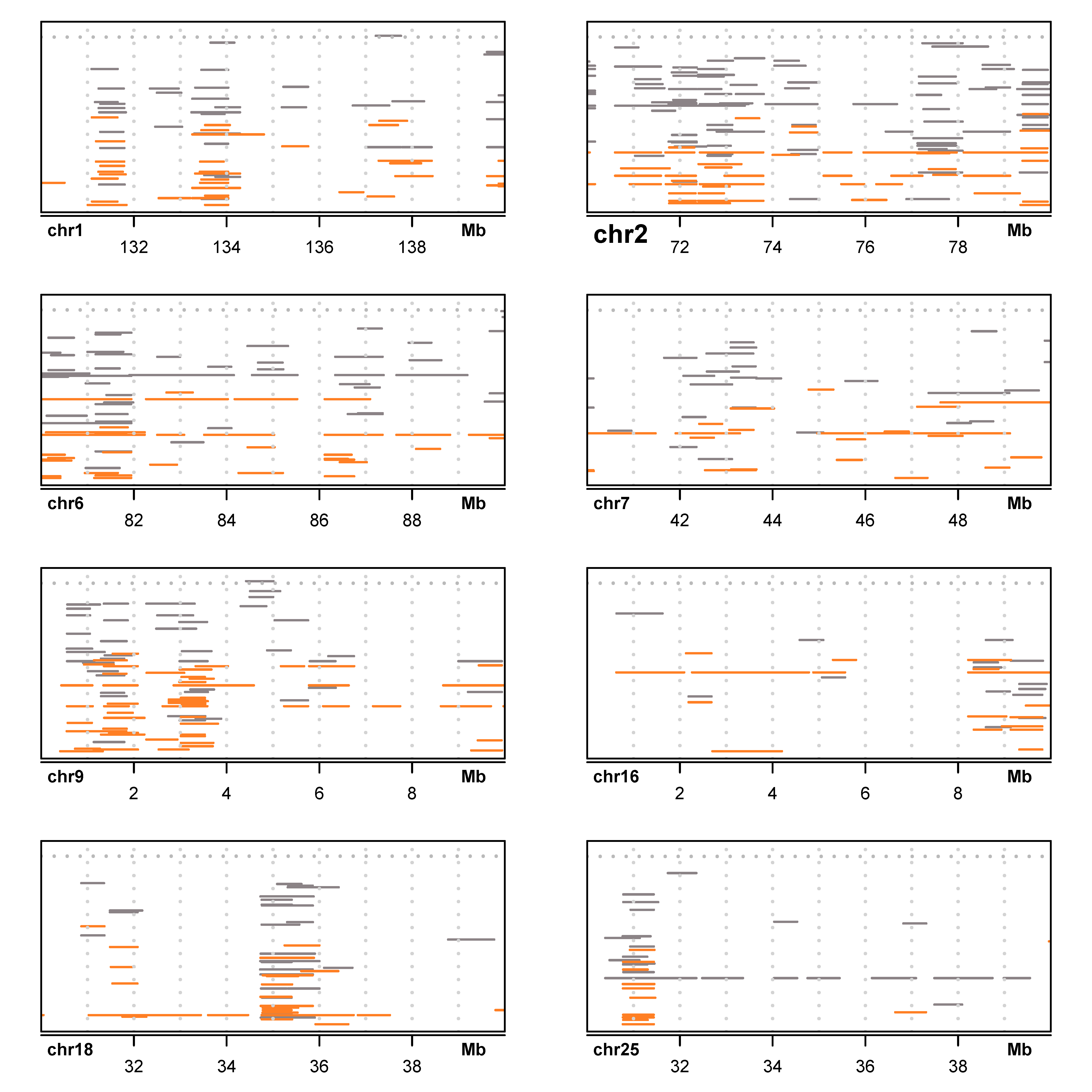

Supplement: S6 Fig — Y axis indicates the individual ID sorted by their KPN number with increasing order, and X axis indicates coordinates on UMD 3.1 reference genome. ROH segments in original dataset (n = 136), and in validation dataset (n = 77) are marked by grey and orange color, respectively. Note that the first 10 individuals is “unselected population” without KPN numbers. (TIF) [file pone.0193701.s007.tif]

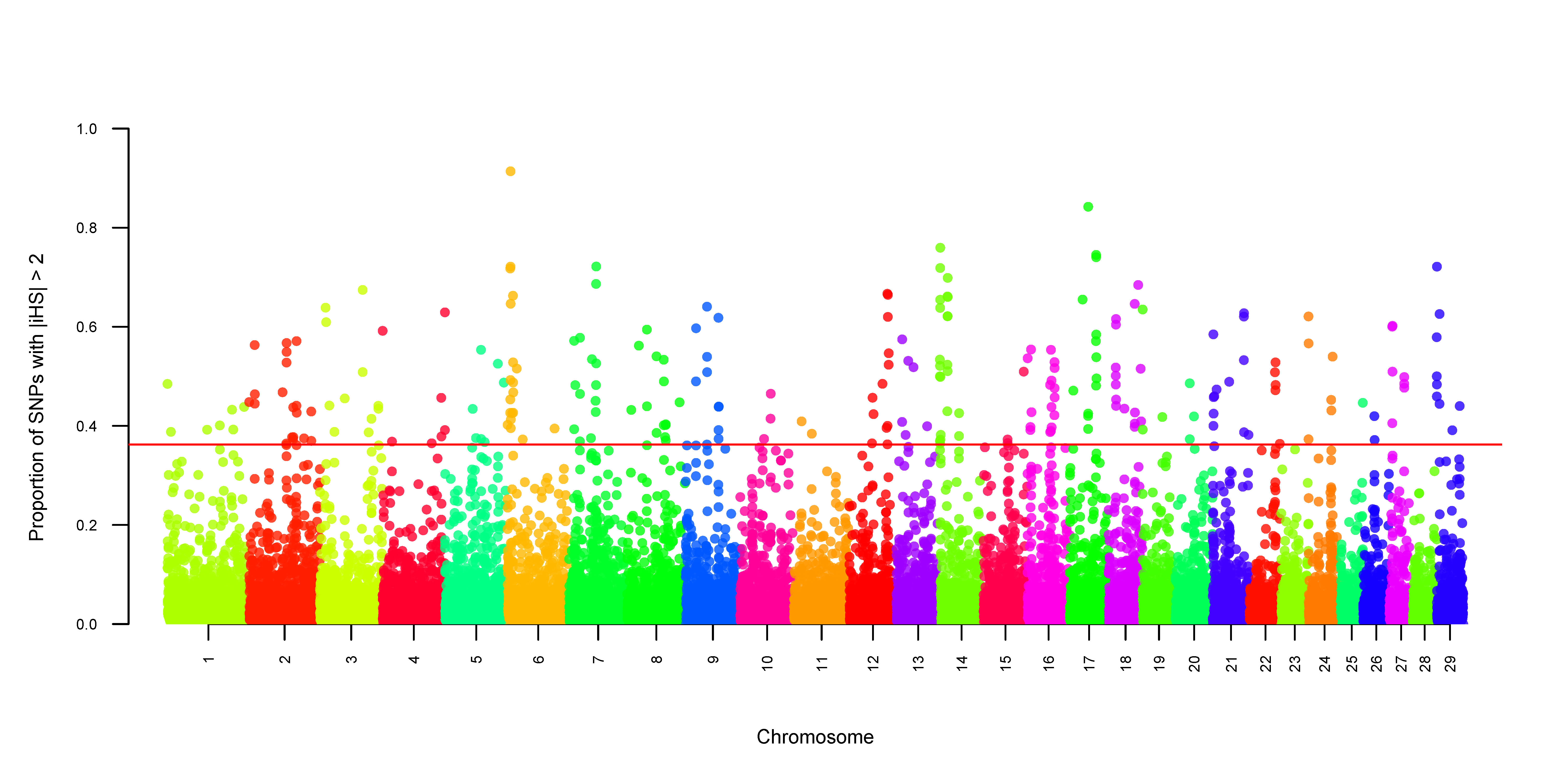

Supplement: S7 Fig — The horizontal red line indicates top 1% proportion of SNPs with |iHS| > 2 in a 100kb window. (TIFF) [file pone.0193701.s008.tiff]
